# Hyperspectral Target Tracking via Spatial–Spectral Attention Weight Variance Gradient and Depth Contrast Enhancement

**DOI:** 10.3390/s26041327

**Published:** 2026-02-19

**Authors:** Yao Yu, Mingkai Ge, Jie Yu, Isaac Kwesi Nooni, Pattathal Vijayakumar Arun, Dong Zhao

**Affiliations:** 1 School of Integrated Circuit Science and Engineering, Wuxi University, Wuxi 214105, China; yyu@cwxu.edu.cn; 2 Jiangsu Province Engineering Research Center of Integrated Circuit Reliability Technology and Testing System, Wuxi University, Wuxi 214105, China; mkge@nuist.edu.cn (M.G.); jyu@stu.cwxu.edu.cn (J.Y.); 3 School of Atmospheric Science and Remote Sensing, Wuxi University, Wuxi 214105, China; 100008@cwxu.edu.cn; 4 School of Computer Science and Engineering Group, Indian Institute of Information Technology, Sri City 441108, India; arun.pv@iiits.in

**Keywords:** hyperspectral video, target tracking, depth contrast enhancement, spatial–spectral attention weight variance gradient, Vision Transformer, scale variation

## Abstract

Scale variations pose a significant challenge in hyperspectral target tracking. To address this challenge, we propose a method that leverages spatial–spectral attention mechanisms combined with depth estimation to enhance the capabilities of the tracker. First and foremost, the method processes raw hyperspectral video inputs through spatial–spectral attention weight variance gradient, utilizing variance gradient for effective dimensionality reduction and obtaining fused spatial–spectral attention weights for subsequent tracking. Moreover, our method integrates a dual-path preprocessing module for handling template and search regions, coupled with a Vision Transformer encoder that incorporates depth contrast enhancement. Last but not least, the proposed tracker is enhanced by the weight adaptive mixed fusion that optimizes the fusion of the fused spatial–spectral attention weights with enhanced depth contrast. The key advantage of our proposed method lies in depth-aware geometric constraints and the use of spectral–spatial information, which enables robust appearance modeling that intrinsically adapts to target scale variations. Extensive experiments on hyperspectral video sequences demonstrate that our method achieves state-of-the-art performance, with an AUC of 0.6704 and a DP@20 of 0.9455, outperforming existing state-of-the-art methods by 3.1% in robustness to scale variations.

## 1. Introduction

Tracking [1,2] the position and motion trajectory of objects has become a critical research area in computer vision (CV), with widespread applications across various domains. Recent advances in hyperspectral sensors technology, coupled with the development of advanced machine learning techniques, have enabled the exploitation of rich spectral information in hyperspectral data. Compared to conventional RGB images, hyperspectral images exhibit greater stability under varying illumination conditions and environmental interference, making hyperspectral target tracking particularly suitable for practical applications such as surveillance [3,4,5], autonomous navigation [6,7,8] and robotics [9,10]. However, despite the advantages of hyperspectral imaging, challenges such as occlusion [11,12], scale variation [13,14,15], and the demand for real-time processing [16,17] continue to hinder accurate target localization.

Recent advances [18] in hyperspectral video (HSV) technologies [19] have facilitated innovative tracking methodologies [20]. Xiong et al. [21] introduced a material-based tracking method, named MHT, utilizing target-specific characteristics. Zhang et al. [22] proposed the use of deep features, in conjunction with spectral matching reduction and adaptive-scale 3D HOG features, to effectively track targets. Li et al. [23] developed SiamBAG, employing band attention grouping and a multibranch network to estimate feature similarity for accurate localization and scale adaptation. SiamHyper approach, proposed by Liu et al. [24], incorporated a hyperspectral target-aware module and a spatial–spectral cross-attention [25] module to improve robustness and address data scarcity. Sun et al. [26] introduced optimizations to streamline hyperspectral image processing, to significantly enhance the computational efficiency of SiamHyper. Tu et al. [27] integrated graph frequency analysis with Beta distribution-based graph wavelets, reformulating hyperspectral anomaly detection as a graph frequency localization problem. Tu et al. [28] combined local convolutional features with global Transformer features for multi-scale background extraction, and introduced a self-attention suppression module (SAS) to inhibit the reconstruction of anomalous pixels. Zhang et al. [29] proposed a spectral–spatial dual-branch fusion transformer (S2DBFT), which leverages weighted fusion and a multi-head spectral–spatial self-attention mechanism to effectively facilitate the interactive learning of spectral and spatial features. Despite these advancements [30], existing methods often struggle with scale variations. Rapid target movements or changes in the camera viewpoint can lead to dynamic scale changes [31], significantly degrading the accuracy of tracking. A novel method, referred to as the spatial–spectral attention and depth information fusion tracker (SSAD-Tracker), is proposed to address these limitations.

The primary motivation of our work is to address the challenges posed by scale variations in hyperspectral target tracking. SSAD-Tracker retains sensitive spectral features to scale to avoid information loss caused by dimension reduction. Meanwhile, the physical relationship between the target depth and the scale is explicitly modeled through the preprocessing module to provide an absolute size reference. Then, dynamic feature fusion is used to adaptively adjust the modal weights according to the complexity of the scene. Finally, through end-to-end joint optimization, the scale-awareness capability is embedded from input to output in the network. SSAD-Tracker not only improves robustness to scale variations but also demonstrates superior generalization capabilities, making it a practical solution for real-world applications.

The main contributions of this paper are threefold as follows.

1. We propose an effective dimensionality reduction method that compresses spectral data while calculating spatial–spectral attention weight variance gradient to ensure real-time performance and retains sensitive spectral features.
2. We propose a multi-modal tracking framework that integrates depth estimation. By adding depth contrast enhancement geometric constraints through multi-modal methods, our tracker achieves better robustness against scale variations.
3. We propose the weight adaptive mixed fusion to optimize the fusion of spatial, spectral, and depth information. This module dynamically adjusts the fusion process based on the relevance of information from each modality.

The paper is structured as follows: Section 2 reviews the research related to the current study, and Section 3 discusses the proposed methodology. Comparative analysis and discussion of the results obtained from the proposed and benchmark HSV-based target tracking algorithms are presented in Section 4. The paper concludes with Section 5, which summarizes the findings of the research.

## 2. Related Works

The current section reviews the existing methods related to the proposed target tracking approach, specifically focusing on depth estimation methods, transformer-based tracking methods and multi-modal feature-level fusion strategies.

### 2.1. Depth Estimation

Depth estimation [32], which is crucial to CV, aims to infer the distance of various objects in the image scene. This section focuses on key developments in deep learning-based monocular depth estimation.which

Pioneering work by Eigen et al. [33] utilized deep neural networks to predict depth from single images, marking a significant departure from traditional stereo vision techniques, yet it suffered from lower resolution depth maps. To enhance the precision of depth estimation, Laina et al. [34] proposed a fully convolutional depth residual network. Wofk et al. [35] explored depth convolution to reduce the inference time of the model. However, the resultant depth maps lacked detail due to the nearest-neighbor interpolation in the decoding module. Miangoleh et al. [36] improved the accuracy of depth estimation models through multi-scale feature fusion [37,38,39]. Jun et al. [40] decomposed metric depth maps into normalized depth maps and scale features, achieving a synergy between computational efficiency and accuracy. Recently, advancements have introduced novel approaches. Li et al. [41] proposed CH3Depth, an efficient and flexible depth foundation model utilizing flow matching for improved accuracy, efficiency, and temporal consistency in both image and video depth estimation. Concurrently, Wu et al. [42] presented GeoDepth, a self-supervised monocular depth estimation framework that models 3D scenes as collections of planes, leading to more accurate and continuous depth maps by exploiting intrinsic geometric representations.

The integration of depth estimation into single target tracking represents a novel advancement in computer vision, enhancing tracking robustness and accuracy by overcoming limitations such as scale changes, occlusions, and complex motions. The approach also facilitates a three-dimensional perspective to traditional two-dimensional tracking approaches, offering a more comprehensive scene understanding.

### 2.2. Transformer Based Tracking Method

CV [43,44,45] focuses on deriving actionable insights from visual data, facilitating machine interpretation. Transformer models, initiated by Vaswani et al. [46], have significantly enhanced the accuracy and reliability of the automated tracking process. Despite the Vision Transformer (ViT) [47] initially managing only single-scale features, advancements like the Swin Transformer [48] have overcome this through multi-scale feature processing. Subsequent developments include feature-enhanced Siamese trackers proposed by Chen et al. [49], feature extraction enhancements of SwinTrack [50], bidirectional feature flow for improved template-search interaction of OSTrack [43], and Siamese network based approach by Bertinetto et al. [51]. Recently, Zhao et al. [52] proposed a dual-branch Siamese network for fusing hyperspectral and RGB data, enhancing the tracking in multi-modal scenarios. Sun et al. [26] developed the CBFF-Net for hyperspectral target tracking, improving localisation and performance. Zhu et al. [53] presented a dynamic network combining spectral and spatial data, optimizing tracking performance across various conditions. However, the data modalities employed in the aforementioned algorithms are overly singular, leading to the tracking algorithms exhibiting significant performance degradation. The issues are frequent when confronted with varying scenes or conditions, as in most real-world application scenarios. Furthermore, a singular data modality can constrain the variety and quantity of features that the algorithm can exploit. At this point, multi-modal data can enhance the accuracy and robustness of the tracking process.

### 2.3. Multi-Modal Feature-Level Fusion Strategies

Multi-modal feature-level fusion [54,55] is a crucial aspect of enhancing the performance of computer vision systems by leveraging the complementary strengths of different data modalities. This section explores seminal works in the field of feature-level fusion in computer vision.

In the realm of deep learning, recent advancements have significantly propelled multi-modal feature-level fusion beyond traditional approaches, particularly by addressing the inherent modality discrepancies. For instance, Geng et al. [56] introduced an Event-based Visible and Infrared Fusion (EVIF) system, which leverages event cameras to overcome challenges like motion blur and extreme lighting conditions in visible-infrared fusion. Their approach emphasizes a multi-task collaborative framework that synergistically processes event data for visible texture reconstruction and infrared image deblurring, demonstrating superior performance in challenging scenarios. Similarly, Zhao et al. [57] proposed CDDFuse, a correlation-driven dual-branch feature decomposition network for multi-modality image fusion, which effectively captures and combines complementary information from different modalities by decomposing features into modality-specific and common components. Zhuang et al. [58] proposed a dual-image dual-local contrast method that employs odd–even decomposition and dual-neighborhood windows, which enhances infrared dim targets, improving detection and real-time performance. Zhu et al. [59] introduced the NERF-C3D, which uses multi-scale fusion and channel attention to optimize density fields and features, boosting NeRF-based 3D detection. Almujally et al. [60] presented a multi-stage scene learning approach, which ultimately achieving holistic scene semantic comprehension through a fully convolutional network. These contemporary methods move beyond simple concatenation or early fusion, employing sophisticated deep learning architectures to learn more robust and discriminative fused representations.

Transformer introduces mechanisms that allow for dynamic and context-aware processing of data, whose attention mechanism enables the model to focus on different parts of the input data, depending on the task at hand. Hence, the advent of transformers has opened new avenues for feature-level fusion strategies in computer vision. The transformer attention weights can be employed for feature-level fusion as they consider the relevance of features to dynamically combine the different modalities. Hence, the model can learn to prefer features from one modality over another based on their utility toward accurate predictions or decisions, enhancing the adaptability of the model to diverse and changing environments.

## 3. Proposed Method

**Figure 1:**
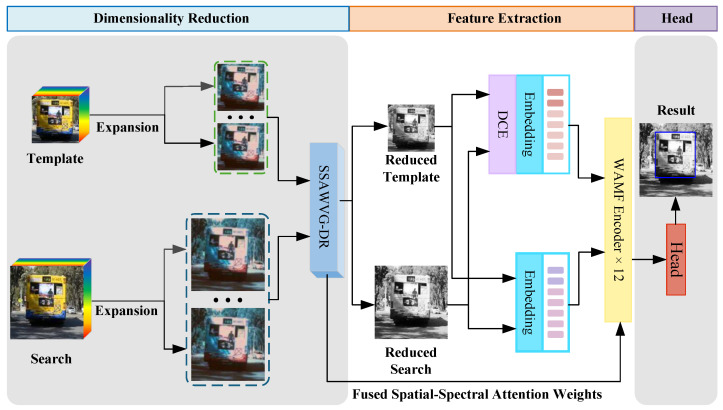
The proposed framework of SSAD-Tracker. Unlike typical hyperspectral tracking frameworks, SSAD-Tracker introduces geometric constraints, utilizes WAMF and integrates the 3D information obtained from depth estimation through DCE.

This section provides a detailed discussion about the proposed SSAD-Tracker framework. Figure 1 summarises the overarching schema of the proposed approach.

This paper presents a novel hyperspectral target tracking framework that addresses scale variation challenges through an integrated multi-modal design. This paper presents a dimensionality reduction technique grounded in spatial–spectral attention weight variance gradients (SSAWVG-DR), which employs variance gradient computations derived from attention weight disparities to compress high-dimensional hyperspectral data into three channels. Next, a dual-stream architecture processes template and search region which called the general processing stream and the fused processing stream. The first stream of the search image or template image is directly preprocessed by the transformer after basic cropping and resizing and then fed to the backbone network. On the contrary, the second stream uses depth contrast enhancement (DCE) preprocessing, is then sent to the transformer preprocessing and is subsequently fed into the backbone network. In the backbone network weight adaptive mixed fusion (WAMF), adaptive mixed fusion is performed on all the features and fused spatial–spectral attention weights. Finally, fully convolutional networks (FCN) are used to further refine the features of the general processing flow search images in order to predict the final bounding box.

We discuss the hyperspectral dimensionality reduction method in Section 3.1. The pre-processing DCE is presented in Section 3.2. The WAMF is introduced in Section 3.3. Finally, the head and loss are summarized in Section 3.4.

### 3.1. Dimensionality Reduction Based on Spatial–Spectral Attention Weight Variance Gradient

The motivation of SSAWVG-DR is to condense the hyperspectral data of the *B* channel into 3 channels to enhance the adaptability to scale variations and improve the computational efficiency. The conventional spatial–spectral attention mechanism in SiamHyper [24] tends to assign high weights due to elevated average responses. In contrast, the variance gradient difference mechanism suppresses uniformly activated but non-discriminative bands, while emphasizing bands with pronounced target–background contrast. This module determines the importance of each band to achieve the best dimensionality reduction accuracy. By retaining the key spectral information, SSAWVG-DR improves the accuracy and reliability of spectral analysis. By reducing the number of spectral channels while retaining scale-sensitive information, SSAWVG-DR reduces the computational burden, improves the adaptability of the framework to scale changes and enhances its generalization ability.

**Figure 2:**
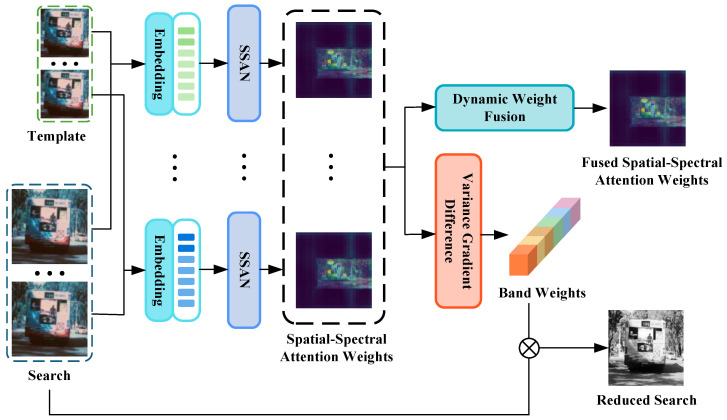
SSAWVG-DR obtains spatial–spectral attention weights through the SSAN, which are then used in the dynamic weight fusion to compute the fused spatial–spectral attention weights for adjusting subsequent tracking. These weights also facilitate the dimensionality reduction results obtained through the variance gradient difference. ⊗ denotes channel-wise weighting.

The template and search images, spanning from the 1st to the *B*-th spectral bands, are first processed through the spatial–spectral attention network (SSAN) on a per-band basis, generating *B* spatial–spectral attention weight tensors. The output of the SSAN is then fed into the dynamic weight fusion and the variance gradient difference. After processing through the dynamic weight fusion, the fused spatial–spectral attention weights are obtained, while the variance gradient difference produces the band weights. The band weights, combined with the original hyperspectral search image, are used to compute a dimension-reduced search image, as shown in Figure 2.

The next step is the spatial–spectral attention network. The target region in the template image after preprocessing by the transformer is denoted as $T\in R^{B\times H_{T}\times W_{T}}$, and the search region in frame *t* after transformer preprocessing is denoted as $S^{t}\in R^{B\times H_{S}\times W_{S}}$. Here, *B* represents the number of spectral bands, and *H* and *W* denote the height and width of the region, respectively. The initial frame estimate of the target region depends on the ground truth from the benchmark dataset.

First, a linear transformation is applied to the tokens formed by *T* and $S^{t}$ through transformer preprocessing to obtain the input data *S*. The normalization of *S* is performed using the following formula:

(1) $$S_{\mathrm{norm}}=\mathrm{LayerNorm}\left(S\right)$$

where LayerNorm(·) represents the Layer Normalization operation.

Then, $S_{\mathrm{norm}}$ is passed through the linear layers of the transformer to obtain the intermediate results $Q_{S}$, $K_{S}$, and $V_{S}$, as given by the following formula:

(2) $$\begin{array}{cc} Q_{S},K_{S},V_{S} & =\mathrm{Transform}(S_{\mathrm{norm}}) \end{array}$$

where Transform(·) denotes the operations within the linear layer of the Transformer that generate the queries, keys, and values.

The spatial–spectral attention weights are computed using the following formula for subsequent processing:

(3) $$\begin{array}{cc} A_{S} & =\mathrm{softmax}(Q_{S}\cdot K_{S}^{T}) \end{array}$$

where softmax(·) denotes the softmax function applied to the input matrix, and *T* represents the matrix transpose operation.

The next step is the dynamic weight fusion. The fused spatial–spectral attention weights, denoted as $W_{FSA}$, are computed using the following formula:

(4) $$\begin{array}{cc} W_{FSA} & =\mathrm{FC}({\mathrm{Concat}}_{b}\left(A_{S}\right)) \end{array}$$

where ${\mathrm{Concat}}_{b}\left(\cdot \right)$ denotes the band-based concatenation operation, and FC(·) represents the fully connected layer.

Then there is the variance gradient difference. The data of $A_{S}$ for the *i*-th spectral band is serialized to obtain $S_{i}$, which represents the serialized result of the *i*-th spectral band data of $A_{S}$. Firstly, the method identifies a subsequence interval for calculating the variance of the lengths of subsequences, subsequently assigning a score based on this variance. Considering computational complexity, this paper uses the same value for left and right. Through experimental validation, it has been determined that setting this value to one-third of the length of $S_{i}$ yields favorable results.

For each possible subsequence length *l* from left to right, we compute the variance of all possible subsequences for each length. For every starting point *j* from 0 to len(S)−l, the variance of the subsequence $S_{i}^{j:j+l}$ is calculated as follows:

(5) $$\begin{array}{c} var_{i}^{j}=\mathrm{Var}\left(S_{i}^{j:j+l}\right) \end{array}$$

It is worth mentioning that although reducing the length of *l* clearly improves accuracy within a certain range, a larger value is not always better. In this experiment, *l* was set to one-third of the total sequence length. Next, we record the subsequence with the minimum variance, denoted by:

(6) $$\begin{array}{c} minVar_{i}=\min\left(var_{i}^{j}\right) \end{array}$$

For each *j*, if $var_{i}^{j}=minVar_{i}$, then for all elements in that subsequence:

(7) $$\begin{array}{c} score\left[j\right]+=1,\mathrm{if}\;var_{i}^{j}=minVar_{i} \end{array}$$

This process is repeated for each *l*, accumulating the scores. We identify the element with the highest score, denoted as MAX=max(score), and output all elements $M_{i}$ that have a score of MAX. If there is more than one such $M_{i}$, the largest $M_{i}$ is selected.

The maximum value of $S_{i}$ is subtracted by $M_{i}$ to obtain $D_{i}$, and the band weight $D_{i}^{\prime }$ is computed using the following formula:

(8) $$\begin{array}{c} D_{i}^{\prime }=\frac{D_{i}-D_{\min}}{D_{\max}-D_{\min}} \end{array}$$

where $D_{\min}$ is the minimum value among all $D_{i}$ values across the bands, $D_{\max}$ is the maximum value among all $D_{i}$ values, and $D_{i}^{\prime }$ is the normalized difference, representing the band weight used for subsequent weighting of the reflectance values in each band.

### 3.2. Depth Contrast Enhancement

The motivation for proposing DCE is to improve the problem of scale variations through depth-scale coupling and resistance to perspective distortion. The variation of the target scale is usually accompanied by a depth variation that is larger near the target and smaller far away. DCE strengthens the depth difference of the target area through deep normalization, enabling the network to directly perceive the actual physical size of the target in real space. Furthermore, when the target approaches or moves away rapidly, the DCE adjusts the depth map to suppress the scale distortion caused by the sudden change in viewing angle.

We employed MiDaS [61] as a pre-trained depth estimation model. And we first rescale the image and forwards it to the depth estimation module using the MiDaS approach. After obtaining the depth map, the depth value at the center of the target, $U_{c}$, normalizes the depth values of the entire image, scaling values below $U_{c}$ down and those above or equal to $U_{c}$ up, creating a depth contrast effect around the target. This process is applied to both the search and template images to produce depth-enhanced renditions. By integrating depth information, DCE improves tracking accuracy, addressing the challenges of scale variation and depth changes that spatial features alone cannot handle. By leveraging depth, the tracker can generate more distinctive target representations, enhancing adaptability to scale fluctuations and focusing on the most relevant regions, ultimately improving robustness and overall performance in real scenes.

**Figure 3:**
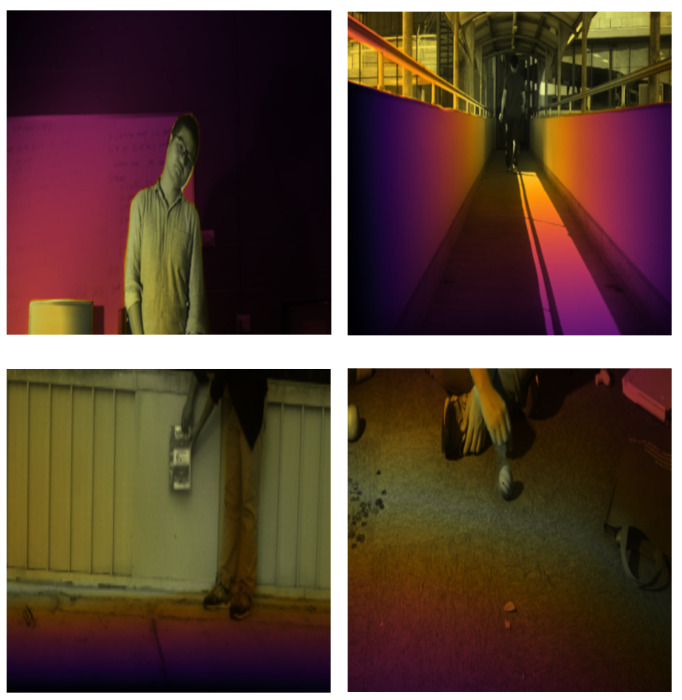
DCE effect on sequence of face2, student, book and ball. The target consistently exhibits a yellow-green hue.

Figure 3 shows a visualization of the effect of DCE, where varying colors within the figure correspond to different depth levels. The DCE ensures that the target maintains a consistent depth, often appearing in a greenish-yellow hue. Importantly, due to the influence of the DCE, unlike conventional depth estimation, there is no direct one-to-one correspondence between depths and colors.

Let $U_{s}\left(x,y\right)$ represent the depth map value at position (x,y) in the search image, and $I_{s}\left(x,y\right)$ denotes the pixel value at the same position. Similarly, for the template image, let $U_{t}\left(x,y\right)$ and $I_{t}\left(x,y\right)$ represent the depth map and pixel values at position (x,y), respectively. The center depth value $U_{c}$ is determined from the bounding box center of the target in the search image. The depth-enhanced images, $I_{s}^{\prime }$ and $I_{t}^{\prime }$, are then obtained as follows:

(9) $$\begin{array}{cc} U_{s}^{\prime }\left(x,y\right) & =\left\{\begin{array}{cc} 0.5⊙\frac{u_{s}\left(x,y\right)}{U_{c}} & \mathrm{if}\;U_{s}\left(x,y\right)<U_{c}\\ 0.5+0.5⊙\frac{U_{s}\left(x,y\right)-U_{c}}{1-U_{c}} & \mathrm{if}\;U_{s}\left(x,y\right)\geq U_{c} \end{array}\right. \end{array}$$

(10) $$\begin{array}{cc} I_{s}^{\prime }\left(x,y\right) & =U_{s}^{\prime }\left(x,y\right)⊙I_{s}\left(x,y\right) \end{array}$$

(11) $$\begin{array}{cc} U_{t}^{\prime }\left(x,y\right) & =\left\{\begin{array}{cc} 0.5⊙\frac{U_{t}\left(x,y\right)}{U_{c}} & \mathrm{if}\;U_{t}\left(x,y\right)<U_{c}\\ 0.5+0.5⊙\frac{U_{t}\left(x,y\right)-U_{c}}{1-U_{c}} & \mathrm{if}\;U_{t}\left(x,y\right)\geq U_{c} \end{array}\right. \end{array}$$

(12) $$\begin{array}{cc} I_{t}^{\prime }\left(x,y\right) & =U_{t}^{\prime }\left(x,y\right)⊙I_{t}\left(x,y\right) \end{array}$$

where the operation ⊙ denotes element-wise multiplication. This process adjusts the depth values based on their relation to $U_{c}$, emphasizing the depth contrast in the vicinity of the target.

The adopted MiDaS methodology utilizes a versatile loss function along with a strategic dataset integration technique. The loss metric is formulated within the parallax domain, ensuring numerical stability and alignment with other relative depth estimation methods. The model consistently exhibits outstanding performance, owing to the scale and shift-invariant loss parameters.

### 3.3. Weight Adaptive Mixed Fusion

**Figure 4:**
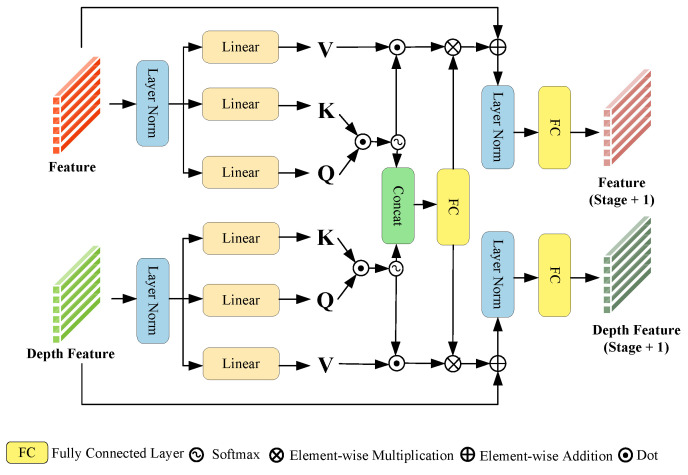
The feature dimension of the weight adaptive mixed fusion encoder is (Batch Size, Number of Patches, Channel).

The motivation for designing WAMF is to effectively integrate multi-modal information using attention weights, as illustrated in Figure 4. Both the search and template images are processed through a Transformer architecture, generating a concatenated feature representation, F, by passing each image through two distinct encoders. The motivation of WAMF is to dynamically fuse multi-modal data while accounting for the varying importance of each modality. Nasir et al. [62] leverage depth information for image compensation and introduce a self-attention mechanism to enhance image details. In contrast, WAMF addresses the imbalanced contribution of appearance and geometric information in object tracking across different scenarios through a dynamic weighting mechanism that assigns attention-based weights to different modalities.

Spatial–spectral features are used to capture apparent changes, such as texture, but they are less sensitive to scale changes. The depth feature provides geometric constraints but has low sensitivity to texture changes. WAMF achieves the joint modeling of appearance and geometry by adaptively weighting both, making the scale estimation more robust. When the target scale undergoes a sudden change, since the depth information directly reflects the actual size of the target, the attention weight of the Depth processing flow will increase accordingly. The general processing flow enhances the scale-sensitive spectral features through fused spatial–spectral attention weights.

The fusion weights of WAMF are learned through a soft attention mechanism in an end-to-end manner. Specifically, WAMF computes adaptive mixed attention weights via a sub-network based on the features of the current frame. These weights directly reflect the reliability and importance of each modality under the given scene context. Furthermore, the fusion weights of WAMF are updated on a per-frame basis; that is, the input of each frame independently passes through the attention weight module to generate the fusion coefficients that best match the current frame. In traditional tracking, relying on a single type of information often limits adaptability to diverse environments. By incorporating attention weights to adjust the fusion process, WAMF enables the tracker to focus on the most relevant features, enhancing robustness, flexibility, and generalization performance across different conditions and modalities.

Similarly, the depth feature, represented as D, is derived from the deeply fused search and template images using analogous operations. Given input features F and depth features D, the process starts with the normalization of input and depth features:

(13) $$F_{norm}=\mathrm{LayerNorm}\left(F\right),\;D_{norm}=\mathrm{LayerNorm}\left(D\right)$$

Subsequently, both $F_{norm}$ and $D_{norm}$ are processed through a transformer linear layer to compute the query Q, key K, and value V matrices:

(14) $$\begin{array}{c} Q_{F},K_{F},V_{F}=\mathrm{Transform}\left(F_{norm}\right),\;Q_{D},K_{D},V_{D}=\mathrm{Transform}\left(D_{norm}\right) \end{array}$$

Attention weights for the features and depth features are computed as follows:

(15) $$\begin{array}{c} A_{F}=\mathrm{softmax}\left(Q_{F}\cdot K_{F}^{T}\right),\;A_{D}=\mathrm{softmax}\left(Q_{D}\cdot K_{D}^{T}\right) \end{array}$$

The intermediate results, $A_{F}^{\prime }$ and $A_{D}^{\prime }$, representing the aggregated information after applying attention, are obtained by:

(16) $$\begin{array}{c} A_{F}^{\prime }=A_{F}\cdot V_{F},\;A_{D}^{\prime }=A_{D}\cdot V_{D} \end{array}$$

The adaptive mixed attention weights $W_{AMF}$ are then computed by concatenating the attention weights of features and depth features and passing them through a fully connected (FC) layer:

(17) $$\begin{array}{c} W_{AMF}=\mathrm{FC}\left(\mathrm{Concat}\left(A_{F},A_{D}\right)\right) \end{array}$$

where Concat(·) denotes the concatenation operation.

The merged features $B_{F}$ and $B_{D}$ are obtained by element-wise multiplication of $W_{AMF}$ with $A_{F}^{\prime }$ and $A_{D}^{\prime }$, followed by addition with the original features:

(18) $$\begin{array}{c} B_{F}=F+\left(A_{F}^{\prime }⊙W_{AMF}\right),\;B_{D}=D+\left(A_{D}^{\prime }⊙W_{AMF}\right) \end{array}$$

Finally, these merged features are normalized and transformed again to produce the output for the current layer of the WAMF module:

(19) $$\begin{array}{c} F_{feature}=\mathrm{FC}\left(\mathrm{LayerNorm}\left(B_{F}\right)\right),\;D_{depth\_feature}=\mathrm{FC}\left(\mathrm{LayerNorm}\left(B_{D}\right)\right) \end{array}$$

The output features $F_{feature}$ and $D_{depth\_feature}$ are then used as inputs for the subsequent layer in the WAMF module sequence.

Specifically, in the first stage, the fused spatial–spectral attention weights, denoted as $W_{\mathrm{FSA}}$, are pixel-wise added to the general attention weights $A_{F}$. The purpose of this operation is to integrate spectral information constraints with the general attention weights, thereby improving the robustness of the tracking process. The operation is represented by the following formula:

(20) $$\begin{array}{c} A_{F}=\mathrm{softmax}\left(Q_{F}\cdot K_{F}^{T}\right)+W_{\mathrm{FSA}} \end{array}$$

### 3.4. Head and Loss

Transforming the padded token sequence of the search area into a two-dimensional spatial feature map marks the initial step. This map is subsequently processed by a Fully Convolutional Network (FCN), comprising a series of *L* Convolution-BatchNorm-ReLU (Conv-BN-ReLU) layers. The output of the FCN encompasses the target classification score map, the local offset matrix, the normalized size parameters. In the target classification score map C, each element $C_{mn}$ in C falls within the range [0, 1] and signifies the probability of target presence at location (m,n). Then, the local offset matrix Δ is designed to correct the spatial quantization error, with $\Delta \in {\left[0,1\right)}^{2\times 4\times \frac{H_{S}}{8}\times \frac{W_{S}}{8}}$, where $H_{S}$ and $W_{S}$ denote the height and width of the search image. Finally, the normalized size parameters Σ represents the bounding box dimensions of the target, scaled to [0, 1].

The precise location of the target, $\left(x^{*},y^{*}\right)$, is pinpointed by locating the peak in C:

(21) $$\begin{array}{c} \left(x^{*},y^{*}\right)={\mathrm{argmax}}_{\left(m,n\right)}C_{mn} \end{array}$$

where the argmax represents the maximization operation.

The definitive bounding box $\left(b_{x},b_{y},b_{w},b_{h}\right)$, encapsulating the target, is deduced as follows:

(22) $$\begin{array}{c} \left(b_{x},b_{y},b_{w},b_{h}\right)=\left(x^{*}+\Delta_{x},y^{*}+\Delta_{y},\Sigma_{w},\Sigma_{h}\right) \end{array}$$

where $\Delta_{x}$ and $\Delta_{y}$ are the offsets, and $\Sigma_{w}$ and $\Sigma_{h}$ denote the width and height from Σ, respectively.

In the training phase, a compound loss function is utilized, merging the weighted focal loss for classification and the combined $\ell_{1}$ and generalized IOU losses for bounding box regression. The composite loss is formalized as:

(23) $$\begin{array}{c} L_{\mathrm{total}}=L_{\mathrm{class}}+L_{\mathrm{bbox}} \end{array}$$

where $L_{\mathrm{class}}$ integrates the focal loss, $L_{\mathrm{bbox}}$ combines the $\ell_{1}$ and IOU losses. A detailed discussion can be found in [63].

## 4. Results and Discussion

We describe the experimental setup in Section 4.1. The datasets used in the experiments are presented in Section 4.2. The evaluation metrics for the experiments are outlined in Section 4.3. A comparison of the trackers is provided in Section 4.4. Qualitative analysis of the experiments is conducted in Section 4.5. Quantitative analysis is presented in Section 4.6. Finally, ablation studies are discussed in Section 4.7.

### 4.1. Experimental Setup

The SSAD-Tracker, as proposed, was realized using PyTorch 1.8.1 with Python 3.7.9, leveraging its powerful capabilities as a Python-based deep learning framework. The experiments, discussed in this study, were conducted on an infrastructure comprising eight NVIDIA Tesla A100 graphics cards, each boasting 40 gigabytes of graphics memory, for training. Testing was performed on a single NVIDIA GeForce RTX 3090 graphics card with 24 gigabytes of graphics memory. Training was facilitated using the AdamW optimizer [64], configured with a batch size of 2, a learning rate of $4\times 10^{-4}$, and a weight decay of $1\times 10^{-4}$. The training was conducted over 300 epochs, ensuring comprehensive learning. Datasets utilized encompassed a diverse set, including GOT-10k [65], COCO [66], LaSOT [67], TrackingNet [68], and the IMEC16 [21] dataset, ensuring thorough evaluation and validation. The proposed model is built upon the ViT-B architecture, specifically employing 12 encoder layers, each with 12 attention heads, and a hidden embedding dimension of 768. In the tracking framework, the template branch and the search branch share weights. The spatial–spectral tokens and depth tokens are embedded via Patch Embedding which patch size is 16 and concatenated along the sequence dimension.

### 4.2. HSV Datasets

**Figure 5:**
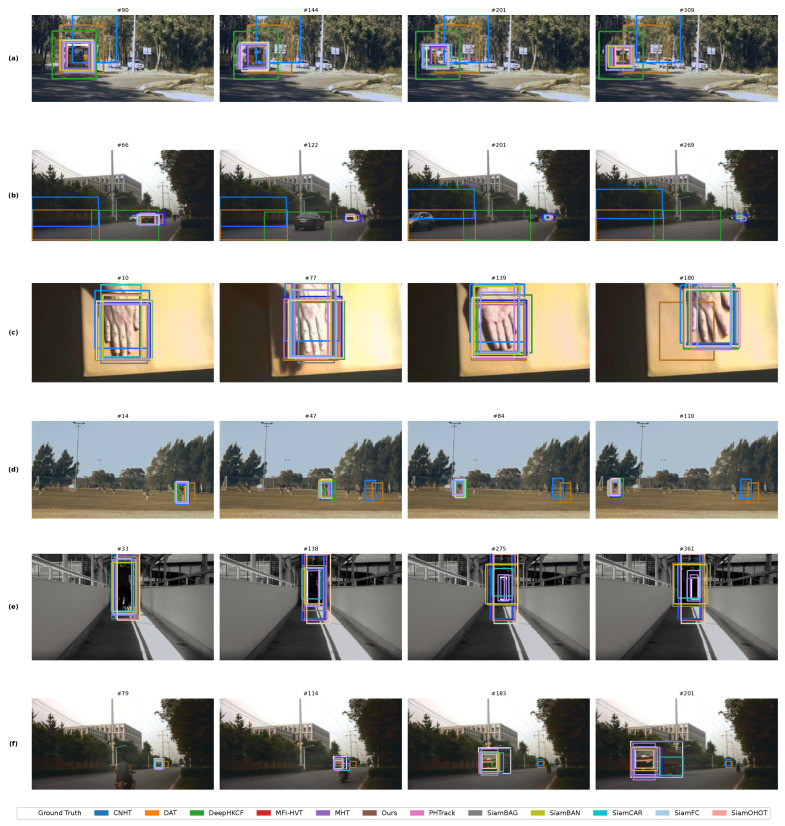
The qualitative analysis of eight trackers on (**a**) bus2; (**b**) car3; (**c**) hand; (**d**) kangaroo; (**e**) student; (**f**) trucker sequences.

The dataset used in this study is available at (http://www.hsitracking.com/) and is provided by the Hyperspectral Object Tracking Challenge (HOTC) [21,69,70], which includes 40 training videos and 35 testing videos. Each set includes both hyperspectral and corresponding visible light videos. In particular, Figure 5a illustrates a bus moving quickly from a near to a far position, with its size varying from 85 × 92 pixels to 61 × 65 pixels. Figure 5b captures two individuals playing basketball, showcasing dynamic movements and occlusions, with their sizes ranging from 20 × 50 pixels to 41 × 111 pixels. Figure 5c depicts a truck advancing along a road from a distant to a closer approach, with its dimensions fluctuating between 17 × 17 pixels and 46 × 43 pixels. Additionally, Figure 5d focuses on the tracking of a worker dressed in red, identified on the rightmost part of the initial frame, amidst two other workers, thereby affecting tracking precision. The hyperspectral visual sequences (HSVs) were captured using a 16-band hyperspectral camera, SnapShot VIS, covering wavelengths from 470 nm to 620 nm with a 10 nm sampling interval. Videos in the dataset are recorded at a frame rate of 25 frames per second (FPS) [23]. The camera is complemented by software capable of adjusting the output image or video to dimensions below the resolution of the camera. The essential characteristics of each video in the test dataset are summarized in Table 1.

This research utilized benchmark sequences outlined in [21] to evaluate the performance of the proposed tracker. Specifically, we chose four representative sequences characterized by scale variation (SV) to assess the effectiveness of SSAD-Tracker. These sequences include bus2, playground, trucker, and worker. Additionally, we incorporated thirty-one sequences presenting challenges such as occlusion (OCC), illumination variation (IV), fast motion (FM), background clutter (BC), out-of-view (OV), and low resolution (LR) to test the robustness and adaptability of SSAD-Tracker across diverse conditions. The proposed method employs a completely identical processing pipeline and model architecture for all challenge attributes. Although the test sequences corresponding to different attributes vary in content and challenging characteristics (e.g., illumination variation, level of occlusion, etc.), the input data undergoes the same preprocessing steps and is processed through a unified network model via forward inference. Therefore, there is no systematic difference in computational complexity or inference time among the attributes, and no additional computational overhead arises from the attributes themselves.

### 4.3. Evaluation Indicators

The current research assesses the efficacy of the proposed tracking method using three quantitative evaluation metrics: success rate (SR), precision rate (PR), and area under the ROC curve (AUC) [21]. The SR, which ranges from 0 to 1, represents the proportion of frames deemed successful where the overlap between the groundtruth and predicted bounding box surpasses a specified threshold. Precision quantifies the accuracy of frames where the predicted location is within a predetermined distance threshold. The distance precision rate at a 20-pixel threshold (DP@20) [21] is also documented. The AUC for each successful instance is utilized as the benchmark for comparing all the tracking methods. It may be noted that the results are compiled based on a one-pass evaluation process.

In the SR graph, the horizontal axis indicates the threshold for overlap, while the vertical axis shows the SR. The formula for calculating the IOU between the groundtruth and the predicted bounding box is given as follows:

(24) $$\mathrm{IOU}=\frac{G_{box}\cap B_{box}}{G_{box}\cup B_{box}}$$

where $G_{box}$ denotes the groundtruth bounding box, and $B_{box}$ represents the predicted bounding box. The symbols ∩ and ∪ stand for the mathematical operations of intersection and union applied to the rectangular boxes, respectively.

The SR quantifies the overlap between the predicted bounding box and the groundtruth using the IOU, defined by the equation:

(25) $$\mathrm{SR}\left(\theta \right)=\frac{N_{\mathrm{IOU}>\theta }}{N_{total}}$$

where SR denotes the success rate, θ is the overlap threshold ranging from 0 to 1, and *N* indicates the number of frames. A frame is deemed successfully tracked when its IOU exceeds the threshold θ.

In the precision graph, the x-axis signifies the Center Location Error (CLE) while the y-axis denotes the PR. CLE measures the discrepancy between the centers of the predicted bounding box and the ground truth, formulated as:

(26) $$\mathrm{CLE}=\sqrt{{\left(x_{pred}-x_{gt}\right)}^{2}+{\left(y_{pred}-y_{gt}\right)}^{2}}$$

where $\left(x_{pred},y_{pred}\right)$ and $\left(x_{gt},y_{gt}\right)$ represent the center coordinates of the predicted bounding box and the groundtruth, respectively. The term CLE quantifies this error.

The PR is quantified by the formula:

(27) $$\mathrm{PR}\left(\sigma \right)=\frac{N_{\mathrm{CLE}<\sigma }}{N_{total}}$$

where PR denotes the precision rate, σ specifies a distance threshold ranging from 0 to 50 pixels, and CLE is the Center Location Error. A frame is recognized as successfully tracked when CLE<σ. Here, $N_{\mathrm{CLE}<\sigma }$ accounts for the count of frames with a center location error smaller than σ, and $N_{total}$ is the total number of frames analyzed.

**Figure 6:**
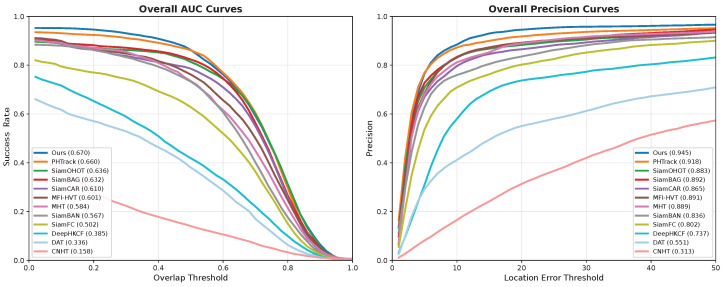
The success rate and precision rate of all sequences.

In target tracking, the precision rate gauges localization accuracy, while the success rate evaluates the fidelity of the bounding box. On the left side of Figure 6, the x-axis represents a range of overlap thresholds from 0 to 1, and the y-axis reflects the success rate, defined as the proportion of frames in which the overlap between the bounding box and the groundtruth surpasses these thresholds. Conversely, in the right segment of Figure 6, the x-axis specifies a range of thresholds for the location error, ranging from 1 to 50 pixels, and the y-axis measures precision. This metric assesses the accuracy of target tracking, computed as the percentage of frames where the tracking error is within the designated location error threshold, indicating successful tracking.

### 4.4. Comparison Methods

The proposed SSAD-Tracker was evaluated against a comprehensive set of state-of-the-art trackers, carefully selected to represent different paradigms and capabilities in both hyperspectral and RGB tracking domains. The comparison includes both classical and contemporary approaches to provide a thorough assessment of SSAD-Tracker performance.

SiamOHOT represents an advanced hyperspectral tracker that extends the Siamese architecture with optimizations crucial for spectral data processing. The hyperspectral target-aware module and spatial–spectral cross-attention make this tracker particularly effective at leveraging the rich spectral information available in hyperspectral imagery. This tracker serves as a primary benchmark for evaluating spectral-specific enhancements and their impact on tracking performance. SiamBAG [23] incorporates a sophisticated band-wise attention that enhances feature extraction from multi-spectral data. The ability to focus on salient spectral features makes this tracker exceptionally relevant for evaluating scale-adaptive tracking across different spectral bands, providing insights into how spectral attention contributes to robust target representation.

MFI-HVT [71] leverages multifaceted feature exploration to improve target separability in complex hyperspectral environments. The approach to feature richness and extraction makes this tracker an ideal comparison point for assessing the effectiveness of multi-modal feature integration strategies employed in SSAD-Tracker. MHT [21] focuses on material attribute discrimination, which proves crucial for distinguishing targets from spectrally similar backgrounds. This capability remains particularly relevant for evaluating how well different trackers handle the unique challenges posed by hyperspectral data, where spectral similarity can lead to tracking failures.

CNHT [72], DAT [73], and DeepHKCF [74] represent foundational approaches in hyperspectral tracking, providing essential baselines for measuring progress in the field. These methods help establish the performance floor and demonstrate the advances achieved by modern approaches. SiamFC [51], SiamCAR [75], SiamBAN [76], and other Siamese-based trackers were included because they represent the dominant paradigm in modern object tracking. SiamFC provides the fundamental Siamese architecture baseline, while SiamCAR and SiamBAN introduce anchor-free designs and advanced feature fusion strategies that are directly relevant to the innovations in SSAD-Tracker.

Modern trackers like TransT [49], STARK [63], OSTrack [43], SeqTrackV2 [77], and Mixformer [78] represent the cutting-edge of transformer-based tracking. These trackers run on the false-color image data of HOTC. Since false-color images are 3-channel, they can be adapted to RGB trackers, so there is no need for retraining. These comparisons remain essential for evaluating how SSAD-Tracker multi-modal fusion approach compares to pure attention-based solutions. PHTrack and other recent hyperspectral trackers provide direct comparisons within the hyperspectral tracking domain, allowing for precise evaluation of the proposed innovations.

The comprehensive tracker selection ensures evaluation across multiple dimensions of spectral processing capability comparing different approaches to handling hyperspectral data, scale handling evaluating various strategies for managing scale variations, feature integration assessing different fusion and attention mechanisms, computational efficiency comparing real-time performance across different architectures, and generalization ability testing robustness across diverse tracking scenarios. This diverse set of comparison methods provides a robust evaluation framework that thoroughly validates the effectiveness of the proposed SSAD-Tracker across different technical paradigms and application scenarios.

### 4.5. Qualitative Comparison

The experimental results of the proposed tracker and comparison trackers across six challenging test sequences are illustrated in Figure 5. These sequences bus2, car3, hand, kangaroo, student, and truck were specifically selected to demonstrate SSAD-Tracker capabilities in handling scale variation challenges while also showcasing the performance across diverse tracking scenarios. In Figure 5, rectangular boxes of different colors represent predictions from different trackers, as indicated by the legend. The white boxes represent ground truth annotations. The proximity of colored boxes to white boxes in both size and position indicates tracking accuracy, with closer matches demonstrating superior performance.

The bus2 sequence presents significant scale variations as the bus moves through the scene. The branches alongside the road create varying illumination conditions, while the bus undergoes substantial size changes. SSAD-Tracker demonstrates exceptional scale adaptation capability, maintaining accurate bounding box estimation throughout the sequence. Unlike several competing methods that struggle with the combined challenges of illumination variation and scale change, SSAD-Tracker depth contrast enhancement and weight adaptive mixed fusion enable robust tracking performance.

The car3 sequence involves complex urban scenarios with multiple vehicles and varying scale conditions. SSAD-Tracker shows superior performance in maintaining target identity despite the presence of similar objects and scale variations. The multi-modal fusion approach proves particularly effective in this cluttered environment, where spectral information provides additional discriminative power beyond RGB features. The hand sequence presents unique challenges with significant deformation and scale changes of the hand target. SSAD-Tracker spatial–spectral attention proves highly effective in tracking the non-rigid hand movements. The tracker maintains accuracy even when the hand undergoes rapid scale variations and partial occlusions, demonstrating the robustness of the proposed approach.

The kangaroo sequence combines scale variation with motion blur and background clutter challenges. SSAD-Tracker successfully tracks the target throughout the sequence, effectively handling the complex interaction between scale changes and environmental factors. The depth information integration proves particularly valuable in maintaining tracking accuracy during rapid movements. The student sequence demonstrates SSAD-Tracker performance in human tracking scenarios with illumination variations and scale changes. The tracker maintains consistent performance across different lighting conditions and scale variations, showcasing the effectiveness of the multi-modal approach in handling real-world tracking scenarios.

The truck sequence presents one of the most challenging scenarios with dramatic scale changes as the truck approaches the camera. SSAD-Tracker excels in this sequence, accurately tracking the target through extreme scale variations that cause other trackers to fail. The dramatic size increase from distant to close proximity demonstrates the superior scale adaptation capabilities of the proposed method.

Across all sequences, SSAD-Tracker demonstrates consistent superiority, particularly in scale variation scenarios. The integration of depth information through DCE, combined with the weight adaptive mixed fusion, enables the tracker to maintain accurate scale estimation and target localization. While some competing trackers show good performance in specific scenarios, SSAD-Tracker provides the most consistent and robust performance across diverse challenging conditions. The qualitative analysis clearly demonstrates that SSAD-Tracker multi-modal approach, specifically designed for scale variation challenges, provides significant advantages over existing methods. The tracker ability to leverage both spectral information and geometric constraints results in superior tracking performance across diverse and challenging scenarios.

### 4.6. Quantitative Comparison

This section presents comprehensive quantitative analysis of SSAD-Tracker performance compared to state-of-the-art tracking methods. The evaluation encompasses overall performance metrics and detailed challenge-specific analysis to provide thorough insights into the tracker capabilities. SSAD-Tracker achieves outstanding performance across all evaluation metrics. The proposed method attains an AUC of 0.6704, DP@20 of 0.9455, demonstrating exceptional tracking accuracy and robust generalization capability. These results represent significant improvements over existing state-of-the-art methods, with SSAD-Tracker ranking first in both primary metrics.

The performance comparison reveals several key insights. PHTrack achieves the second-best performance with AUC of 0.6602 and DP@20 of 0.9180, representing a strong baseline in hyperspectral tracking. The comprehensive results are detailed in Table 2, while Figure 6 illustrates the success and precision rates across all tracking sequences. Among traditional hyperspectral trackers, SiamOHOT shows competitive performance with AUC of 0.6356 and DP@20 of 0.8828, while classical methods like CNHT and DAT demonstrate significantly lower performance, highlighting the advances achieved by modern deep learning approaches.

The challenge-specific analysis reveals important performance characteristics across different tracking scenarios.

Challenge-specific performance analysis demonstrates the superior adaptability of SSAD-Tracker across various tracking scenarios. The detailed AUC comparison across different challenges in Table 3 reveals the exceptional performance of the tracker in handling diverse tracking conditions. SSAD-Tracker achieves the highest AUC of 0.6763 and DP@20 of 0.9285 in scale variation challenges, directly validating the core contribution of this work. The proposed spatial–spectral attention attention weight variance gradient and depth contrast enhancement enable superior scale adaptation compared to existing methods.

The tracker ranks first in occlusion challenges with AUC of 0.6467 and DP@20 of 0.9201, demonstrating the effectiveness of multi-modal fusion in maintaining target identity during occlusion events. The integration of spectral and spatial information provides robustness against partial occlusions. The corresponding DP@20 performance metrics across various challenges are presented in Table 4, which further validates the consistent superiority of the tracker.

To provide a more comprehensive evaluation, we expanded the comparison to include additional state-of-the-art trackers across different paradigms. The extended AUC comparison in Table 5 demonstrates SSAD-Tracker competitive advantages against both traditional and modern tracking approaches. SSAD-Tracker achieves outstanding performance in OPR scenarios with AUC of 0.7574 and DP@20 of 0.9699, ranking first in both metrics. This superior performance indicates the tracker capability to handle complex geometric transformations through the weight adaptive mixed fusion.

The tracker demonstrates excellent performance in illumination variation with AUC of 0.6238 and DP@20 of 0.9097 and various motion-related challenges. The depth contrast enhancement module proves particularly effective in handling illumination changes while maintaining tracking accuracy. The extended DP@20 evaluation results in Table 6 further confirm robust performance of the tracker across the expanded set of comparison methods.

Under the SV attribute, our method, which integrates spatial–spectral attention mechanisms with depth information, demonstrates enhanced robustness and tracking accuracy compared to other algorithms with AUC of 0.6763 and DP@20 of 0.9285.

While SSAD-Tracker shows strong overall performance, some challenges present opportunities for future enhancement. In background clutter scenarios, the tracker achieves good but not optimal performance, suggesting potential for architectural improvements in handling complex backgrounds. The comprehensive comparison reveals SSAD-Tracker advantages across different paradigms. SSAD-Tracker outperforms dedicated hyperspectral trackers like SiamOHOT and MHT, demonstrating the effectiveness of the proposed multi-modal fusion approach.

Compared to recent transformer approaches like SeqTrackV2 and Mixformer, SSAD-Tracker shows competitive or superior performance while maintaining computational efficiency. The tracker significantly outperforms classical Siamese methods, validating the importance of multi-modal processing and single-stream ViT networks. The quantitative analysis conclusively demonstrates SSAD-Tracker state-of-the-art performance in hyperspectral object tracking, particularly excelling in scale variation scenarios while maintaining robust performance across diverse challenging conditions. The tracker ability to achieve superior accuracy while maintaining real-time performance makes the approach highly suitable for practical applications.

### 4.7. Ablation Study

The components evaluated include DCE (depth contrast enhancement), which enhances tracking by adding depth information; WAMF (weight adaptive mixed fusion), which adapts and combines features from multiple modalities; and *l divide*, a method that divides the difference sequence $S_{i}$ based on length *l*, determined by the sequence length division. When *l divide* is set to NS, the fused spatial–spectral attention weights are not used. If *l divide* is set to PCA, it indicates the use of principal component analysis (PCA) [79] as the dimensionality reduction method for hyperspectral images.

The performance of each setup is summarized in Table 7. The baseline model, which does not use the DCE or WAMF, demonstrates good efficiency, achieving an AUC of 0.6309 and a DP@20 of 0.8408, with a high FPS of 67.2. However, despite its efficiency, this setup shows considerable room for improvement in terms of tracking accuracy. The lack of depth information and feature adaptation limits its ability to handle complex tracking scenarios effectively.

When only the DCE module is introduced, the model experiences a decline in performance, with AUC dropping to 0.6013 and DP@20 reducing to 0.8316, reflecting a decrease of 6.91% and 11.39%, respectively. The FPS also decreases significantly to 28.2. These results suggest that DCE is not a generic performance-enhancement module but rather a geometric scale correction tool. Its primary function is to normalize scale variations induced by depth changes and to mitigate perspective distortion, rather than to directly enhance the discriminative power of appearance features. Concretely, DCE emphasizes physical scale consistency through depth normalization and rescaling, which is beneficial for geometric modeling; however, it may also alter the statistical distribution of features, thereby weakening discriminative cues that are sensitive to texture and appearance details—particularly in scenarios where appearance information is more reliable than depth information. Consequently, when DCE is used alone, depth noise and distribution distortion can lead to a decline in accuracy. In contrast, when combined with WAMF, the contribution of depth information is adaptively amplified only when it is truly beneficial, resulting in improved overall tracking performance. Additionally, the computational overhead caused by the DCE substantially reduces processing speed, indicating that its benefits come with trade-offs in efficiency.

In contrast, when the WAMF is used independently, there is a noticeable improvement in performance. The model achieves an AUC of 0.6615 and DP@20 of 0.9374, both showing significant improvements of 3.06% and 9.66% respectively compared to the baseline. The FPS, however, decreases to19.2, which is a trade-off for the enhanced tracking accuracy. The results indicate that This module dynamically adjusts the weights of spatial–spectral features and depth features, adaptively fusing information based on the scene, enhancing robustness to scale variations.

Further analysis of the impact of the sequence division parameter reveals that different values of *l divide* influence both tracking accuracy and computational efficiency. The results show that setting *l divide* to 2 or 4 yields stable performance, with slight variations in both AUC and DP@20. Specifically, when *l* divide=2, AUC is 0.6632, and when *l* divide=4, AUC reaches 0.6684. However, setting *l divide* to NS results in a sharp reduction in SV performance, with AUC dropping to 0.6622 and DP@20 decreasing to 0.9279. This configuration, however, leads to a slight increase in FPS because the fusion step is skipped, reducing the computational complexity.

However, when PCA is used for dimensionality reduction *l* divide=PCA, we observe a slight reduction in tracking performance compared to *l* divide=3. The AUC drops slightly to 0.6642, and DP@20 decreases to 0.9341, representing a decrease of 0.62% and 1.14% respectively. This clearly demonstrates that our dimensionality reduction algorithm achieves effective dimensionality reduction by calculating the variance gradient of the spatial–spectral attention weights. It effectively separates the background from the target while retaining the key information related to the scale change of the target.

Overall, the results from this ablation study highlight that the combination of DCE, WAMF, an appropriate sequence division strategy and the use of the fused spatial–spectral attention weights provides the most balanced performance. While PCA provides a more efficient approach in terms of speed, the *l* divide=3 setting offers the best tracking performance in terms of both accuracy and efficiency. While the DCE alone does not significantly enhance accuracy and reduce FPS, WAMF improves tracking performance considerably. The choice of *l divide* value and the use of the fused spatial–spectral attention weights further fine-tune the trade-off between accuracy and processing speed, with *l* divide=3 providing the best balance. While PCA provides a more efficient approach in terms of speed, the *l* divide=3 setting offers the best tracking performance in terms of both accuracy and efficiency.

As shown in Table 8, when introducing DCE alone, the AUC decreased by 3.17% and 4.29% under the OPR and OV attributes, respectively. This degradation is attributed to depth noise and distribution distortion, which compromise tracking accuracy when DCE is utilized in isolation.

When employing WAMF alone, the AUC increased by 7.12% and 4.72% under the OPR and OV attributes, respectively. This enhancement stems from the weight adaptive mixed fusion module, which adaptively integrates multi-modal features to construct a more robust and discriminative feature representation for both the target and its surrounding context.

When SSAWVG-DR is replaced with PCA, and both DCE and WAMF are incorporated, the AUC increased by 10.34% and 9.78% under the OPR and OV attributes, respectively. This is because DCE, as a geometric scale calibration tool, suffers from depth noise and distribution distortion when used independently, leading to limited tracking accuracy. However, when integrated with the WAMF module, the mechanism dynamically amplifies the weight of depth information at critical discriminative moments, thereby substantially enhancing tracking performance. Meanwhile, the variance gradient difference mechanism suppresses spectrally homogeneous yet weakly discriminative frequency bands while emphasizing bands with significant target–background contrast.

We employ a dual-path preprocessing structure with the principal objective of incorporating the Depth Contrast Enhancement (DCE) module, thereby systematically addressing the challenges of scale variation and depth perception that are difficult to resolve when relying exclusively on spatial appearance features. To validate the rationality of this architectural choice, we conduct a direct comparison in Table 7 of the main manuscript. Experimental results demonstrate that the dual-path architecture integrated with the DCE module (i.e., the complete “Ours” model in Table 7) achieves significant improvements across multiple evaluation metrics, including AUC and DP@20, compared to the unified single-path feature extractor (i.e., the Baseline in Table 7).

In conclusion, the integration of the DCE and WAMF module, the careful selection of *l divide* and the use of the fused spatial–spectral attention weights enable a highly effective and efficient tracking model. The findings indicate that while DCE helps improve contextual understanding, the fusion of features through WAMF is crucial for achieving superior tracking performance.

## 5. Conclusions

This paper proposes a dimensionality reduction method based on spatial–spectral attention weight variance gradients, which significantly enhances the target tracking process. The proposed method reduces the dimensionality of hyperspectral data, improves the separability between the target and background, and generates fused spatial–spectral attention weights. By incorporating depth information from 3D scenes and utilizing the WAMF module, the framework effectively integrates multi-modal data to improve tracking robustness. Experimental results demonstrate that this approach achieves high tracking accuracy, with an AUC of 0.6704 and DP@20 of 0.9455, outperforming existing state-of-the-art methods.

However, despite its promising performance, the proposed method has some limitations. While the tracker performs well in handling target scale variations, its ability to manage large amounts of background clutter still requires improvement. Although the integration of spectral and depth information enhances performance, further optimization is needed to handle scenarios with significant background interference. Additionally, while the SSAD-Tracker excels at adapting to scale fluctuations, further research is required to better balance its robustness to background clutter and its overall performance in more complex environments.

In the future, efforts will be directed towards improving the handling of background clutter of the method by refining the fusion of spectral and depth information. WAMF can be applied to multi-target tracking tasks. By enhancing the fusion of spatial–spectral and depth information among different targets, it improves the robustness in complex scenarios. Furthermore, the application of WAMF in dynamic real environments can focus on autonomous driving and unmanned aerial vehicle (UAV) vision tasks, where the target sizes and environmental changes are significant, and it is necessary to quickly adapt to different visual information.

## Figures and Tables

**Table 1:** The details of experiment sequences.

Sequences	Frames	Size	Challenge
*ball*	625	471 × 207	MB, OCC, SV
*basketball*	186	463 × 256	FM, LR, OCC, MB
*board*	471	277 × 140	IPR, OCC, BC, OPR, SV
*book*	601	317 × 148	IPR, DEF, OPR
*bus*	131	261 × 148	LR, FM, BC
*bus2*	326	361 × 167	IV, SV, FM, OCC
*campus*	976	384 × 157	IV, OCC, SV
*car*	101	261 × 148	SV, IPR, OCC, OPR
*car2*	131	351 × 167	SV, OPR, IPR,
*car3*	331	512 × 256	SV, OCC, LR, IV
*card*	930	363 × 200	IPR, OCC, BC
*coin*	149	291 × 120	BC
*coke*	731	493 × 207	BC, IPR, FM, OPR, SV
*drive*	725	297 × 142	BC, IPR, OPR, SV
*excavator*	501	500 × 240	IPR, SV, OCC, OPR, DEF
*face*	279	446 × 224	IPR, SV, OPR, MB
*face2*	1111	446 × 224	IPR, SV, OPR, OCC
*forest*	530	512 × 256	BC, OCC
*forest2*	363	512 × 256	BC, OCC
*fruit*	552	493 × 232	BC, OCC
*hand*	184	314 × 186	SV, BC, DEF, OPR
*kangaroo*	117	385 × 206	SV, BC, DEF, OPR, MB
*paper*	278	446 × 224	IPR, BC
*pedestrian*	306	351 × 167	IV, SV
*pedestrian2*	363	512 × 256	OCC, LR, DEF, IV
*player*	901	463 × 256	IPR, OPR, DEF, SV
*playground*	800	463 × 256	SV, OCC
*rider1*	336	512 × 256	LR, IV, OCC, SV
*rider2*	210	512 × 256	LR, IV, OCC, SV
*rubik*	526	493 × 207	DEF, IPR, OPR
*student*	396	438 × 256	IV, SV
*toy1*	376	271 × 135	BC, OCC
*toy2*	601	371 × 171	BC, IV, OCC, SV, OPR
*truck*	221	512 × 256	OCC, SV, IV, OV
*worker*	1209	228 × 121	SV, LR, BC

**Table 2:** The overall performance of various trackers is compared on RGB and hyperspectral/false-color videos, with the best and second-best values highlighted in red and *blue* fonts, respectively.

Tracker	AUC	DP@20	FPS
CNHT	0.1580	0.3134	2.82
DAT	0.3362	0.5507	*124.8*
DeepHKCF	0.3851	0.7372	1.02
MFI-HVT	0.6008	0.8914	6.3
MHT	0.5843	0.8894	7.6
PHTrack	*0.6602*	*0.9180*	15.2
SiamBAG	0.6323	0.8921	5.7
SiamBAN	0.5671	0.8357	63.1
SiamCAR	0.6098	0.8653	64.2
SiamFC	0.5021	0.8025	189
SiamOHOT	0.6356	0.8828	*36.8*
Ours	0.6704	0.9455	19.2

**Table 3:** AUC comparison across different challenges, with the best and second-best values highlighted in red and *blue* fonts, respectively.

Tracker	BC	DEF	FM	IV	IPR	LR	MB	OCC	OPR	OV	SV
MFI-HVT	0.6514	0.6391	0.5996	0.5154	0.6924	0.5136	0.5701	0.5459	0.6804	0.6107	0.5990
MHT	0.6263	0.6486	0.5673	0.5047	0.6889	0.4263	0.5580	0.5620	0.6937	0.6243	0.5817
SiamBAG	0.6479	0.6915	0.6146	0.5337	0.7034	0.5829	0.6499	0.5970	0.6828	0.6346	0.6223
SiamBAN	0.5365	0.6629	0.6115	0.4913	0.6478	0.5414	*0.6693*	0.5279	0.6632	0.6292	0.5801
SiamCAR	0.6226	*0.7172*	0.6611	0.5279	0.6746	0.5643	0.6634	0.5781	0.6903	*0.6390*	0.5932
SiamOHOT	0.7003	0.7164	0.5621	0.5185	*0.7332*	0.5000	0.6115	0.5566	0.7291	0.5842	0.6282
SiamFC	0.4832	0.6329	0.6320	0.4020	0.5896	0.4707	0.5833	0.5301	0.6128	0.4575	0.4835
PHTrack	*0.6784*	0.6743	0.7349	*0.5778*	0.7250	*0.5820*	0.7300	*0.6176*	*0.7087*	0.6035	*0.6462*
DeepHKCF	0.4264	0.5426	0.3772	0.1731	0.5493	0.1873	0.4112	0.3122	0.5210	0.3122	0.3647
DAT	0.4186	0.3669	0.2819	0.1711	0.4342	0.2776	0.3246	0.2913	0.4004	0.2522	0.2768
CNHT	0.1653	0.2389	0.1805	0.0994	0.2387	0.0313	0.1182	0.1257	0.2386	0.1440	0.1634
Ours	0.6758	0.7304	*0.6879*	0.6238	0.7517	0.6027	0.6685	0.6467	0.7574	0.8452	0.6763

**Table 4:** DP@20 comparison across different challenges, with the best and second-best values highlighted in red and *blue* fonts, respectively.

Tracker	BC	DEF	FM	IV	IPR	LR	MB	OCC	OPR	OV	SV
MFI-HVT	0.9290	0.8851	0.8322	*0.8800*	0.9519	0.8726	0.8414	0.8118	0.9505	0.8959	0.9134
MHT	*0.9499*	0.9007	0.8419	0.8298	*0.9647*	0.8011	0.8442	0.8120	*0.9641*	0.8507	0.8955
SiamBAG	0.8988	0.9357	0.8831	0.8415	0.9301	0.8377	0.8987	0.8292	0.9056	0.8597	0.8905
SiamBAN	0.7845	0.9055	0.9659	0.7819	0.8991	0.8751	0.9817	0.7857	0.9218	0.8507	0.8656
SiamCAR	0.8641	*0.9377*	0.9509	0.8233	0.8929	0.8822	0.9328	0.8082	0.9092	0.8552	0.8670
SiamOHOT	0.9303	0.9348	0.8161	0.8204	0.9598	0.7794	0.8186	0.7974	0.9594	0.8552	0.8926
SiamFC	0.7560	0.9252	0.9645	0.7636	0.8564	0.8514	0.9317	0.8185	0.8848	0.8507	0.7934
PHTrack	0.9250	0.9193	*0.9926*	0.8633	0.9516	*0.8913*	0.9865	*0.8799*	0.9466	0.8552	*0.9138*
DeepHKCF	0.7847	0.8202	0.8747	0.5540	0.8461	0.5631	0.8517	0.6014	0.8365	*0.9186*	0.7510
DAT	0.5897	0.4625	0.4023	0.3597	0.7027	0.4820	0.6023	0.4720	0.6011	0.5204	0.4727
CNHT	0.2780	0.4528	0.3468	0.2482	0.4212	0.1035	0.2750	0.2107	0.4075	0.3801	0.2996
Ours	0.9588	0.9669	0.9960	0.9097	0.9852	0.9206	*0.9844*	0.9201	0.9699	1.0000	0.9285

**Table 5:** AUC comparison across different challenges after expanding trackers, with the best and second-best values highlighted in red and *blue* fonts, respectively.

Tracker	BC	DEF	FM	IPR	IV	LR	MB	OCC	OPR	OV	SV
CNHT	0.1653	0.2389	0.1805	0.2387	0.0994	0.0313	0.1182	0.1257	0.2386	0.1440	0.1634
MHT	0.6263	0.6486	0.5673	0.6889	0.5047	0.4263	0.5580	0.5620	0.6937	0.6243	0.5817
MFI-HVT	0.6514	0.6391	0.5996	0.6954	0.5154	0.5136	0.5701	0.5459	0.6804	0.6107	0.5990
DeepHKCF	0.4264	0.5426	0.3772	0.5493	0.1731	0.1873	0.4112	0.3122	0.5210	0.3122	0.3647
DAT	0.4186	0.3669	0.2819	0.4342	0.1711	0.2776	0.3246	0.2913	0.4004	0.2522	0.2768
SiamBAN	0.5365	0.6629	0.6115	0.6478	0.4913	0.5414	0.6693	0.5279	0.6632	0.6292	0.5801
SiamCAR	0.6226	0.7172	0.6611	0.6746	0.5279	0.5643	0.6634	0.5781	0.6903	0.6390	0.5932
SiamFC	0.4832	0.6329	0.6320	0.5896	0.4020	0.4707	0.5833	0.5301	0.6128	0.4575	0.4835
SiamGAT	0.5110	0.6880	0.6750	0.6270	0.5580	0.5760	0.6870	0.5910	0.6470	0.6010	0.5860
TransT	0.5960	0.7240	0.6310	*0.7250*	0.5540	0.5500	0.6790	0.6060	0.7290	0.7800	0.6310
STARK	0.5550	0.7020	0.6130	0.6910	*0.5820*	0.5870	0.6550	0.6120	0.6990	0.8070	0.6040
OSTrack	0.6310	0.7200	0.6720	0.7240	0.5800	0.5390	0.6630	0.6260	*0.7400*	0.8370	0.6410
SeqTrackV2	0.6270	0.7300	*0.6930*	0.7140	0.6320	0.6280	0.6820	0.6610	0.7180	0.8210	*0.6610*
Mixformer	0.6200	0.7530	0.6440	0.7170	0.5710	0.5740	0.6480	0.6350	0.7120	0.8470	0.6040
SiamBAG	0.6479	0.6915	0.6146	0.7034	0.5337	0.5829	0.6499	0.5970	0.6828	0.6346	0.6223
SiamOHOT	0.7003	0.7164	0.5621	0.7332	0.5185	0.5000	0.6115	0.5566	0.7291	0.5842	0.6282
Trans-DAT	0.4470	0.6260	0.5880	0.6160	0.5150	0.4710	0.5570	0.5040	0.6110	0.7740	0.5410
PHTrack	*0.6784*	0.6743	0.7349	*0.7260*	0.5790	0.5820	0.7340	0.6190	0.7087	0.6035	0.6470
Ours	0.6758	*0.7304*	0.6879	0.7620	0.6238	*0.6027*	*0.7250*	*0.6467*	0.7574	*0.8452*	0.6763

**Table 6:** DP@20 comparison across different challenges after expanding trackers, with the best and second-best values highlighted in red and *blue* fonts, respectively.

Tracker	BC	DEF	FM	IPR	IV	LR	MB	OCC	OPR	OV	SV
CNHT	0.2770	0.4440	0.3490	0.2390	0.2470	0.1040	0.2770	0.2100	0.4030	0.3710	0.2980
MHT	*0.9499*	0.9007	0.8419	*0.9647*	0.8300	0.8011	0.8442	0.8120	*0.9641*	0.8510	0.8955
MFI-HVT	0.9290	0.8851	0.8322	0.9520	0.8790	0.8726	0.8414	0.8118	0.9505	0.8959	0.9134
DeepHKCF	0.7850	0.8202	0.8747	0.8470	0.5530	0.5620	0.8530	0.6014	0.8370	0.9190	0.7510
DAT	0.5897	0.4625	0.4023	0.7027	0.3597	0.4820	0.6023	0.4720	0.6011	0.5204	0.4727
SiamBAN	0.7845	0.9055	0.9659	0.8991	0.7819	0.8751	0.9820	0.7857	0.9218	0.8507	0.8656
SiamCAR	0.8641	0.9377	0.9509	0.8929	0.8233	0.8822	0.9328	0.8082	0.9092	0.8552	0.8670
SiamFC	0.7560	0.9252	0.9645	0.8564	0.7636	0.8514	0.9317	0.8185	0.8848	0.8507	0.7934
SiamGAT	0.7110	0.9590	0.9070	0.8230	0.8270	0.8480	0.9900	0.8230	0.8800	0.8640	0.8360
TransT	0.8350	0.9360	0.9460	0.9470	0.8360	0.8240	0.9910	0.8480	0.9460	0.9770	0.8890
STARK	0.8080	*0.9740*	0.9230	0.9270	0.8650	0.8890	0.9370	0.8770	0.9360	0.9950	0.8530
OSTrack	0.8720	0.9260	0.9750	0.9330	0.8350	0.7740	0.9440	0.8640	0.9570	0.9950	0.8850
SeqTrackV2	0.8640	0.9420	0.9920	0.8910	*0.8910*	0.9250	0.9720	*0.9050*	0.9150	*1.0000*	0.9000
Mixformer	0.8700	0.9770	0.9640	0.9460	0.7840	0.8110	0.9260	0.8710	0.9300	0.9910	0.8220
SiamBAG	0.8988	0.9357	0.8831	0.9301	0.8415	0.8377	0.8987	0.8292	0.9056	0.8597	0.8905
SiamOHOT	0.9303	0.9348	0.8161	0.9598	0.8204	0.7794	0.8186	0.7974	0.9594	0.8552	0.8926
Trans-DAT	0.6670	0.8690	0.9440	0.8350	0.7460	0.7490	0.8990	0.7320	0.8310	0.9820	0.7770
PHTrack	0.9250	0.9193	*0.9940*	0.9520	0.8633	0.8913	*0.9865*	0.8799	0.9470	0.8552	*0.9140*
Ours	0.9588	0.9669	0.9960	0.9952	0.9097	*0.9206*	0.9844	0.9201	0.9699	1.0000	0.9285

**Table 7:** Ablation Study of SSAD-Tracker on Benchmark and SV Sequences.

Experiment	AUC	DP@20	AUC (SV)	DP@20 (SV)	FPS
**Baseline (No DCE, No WAMF)**	0.6309	0.8408	0.6126	0.8329	67.2
**DCE Only (No WAMF)**	0.6013	0.8316	0.6013	0.8221	28.2
**WAMF Only (No DCE)**	0.6615	0.9374	0.6675	0.9374	19.2
**DCE + WAMF, *l*** divide=2	0.6632	0.9332	0.6361	0.9064	19.2
**DCE + WAMF, *l*** divide=3	0.6704	0.9455	0.6745	0.9455	19.2
**DCE + WAMF, *l*** divide=4	0.6684	0.9424	0.6694	0.9455	19.2
**DCE + WAMF, *l*** divide=NS	0.6622	0.9279	0.6292	0.8944	19.7
**DCE + WAMF, *l*** divide=PCA	0.6642	0.9341	0.6456	0.9216	19.7

**Table 8:** Ablation Study(AUC) of each component on OPR and OV Sequences.

Methods	Baseline	Only D	Only W	D + W, *l* divide=PCA	D + W, *l* divide=3
OPR	0.6458	0.6253	0.6918	0.7126	0.7574
OV	0.6827	0.6534	0.7149	0.7495	0.8452

D denotes the DCE module, and W denotes the WAMF module. Both complex geometric transformations and viewpoint changes are subsumed under the OPR and OV attributes.

## Data Availability

The raw data supporting the conclusions of this article will be made available by the authors on request.
